# COVID-19 Lab: A Whistlestop Journey at a Tertiary Health Care Center

**DOI:** 10.7759/cureus.10162

**Published:** 2020-08-31

**Authors:** Sulin K Behera, Soumyashree Mohapatra, Dipankar Pattnaik, Swetalina Jena, Satyabrata Thakur, Sumanta Sahu

**Affiliations:** 1 Microbiology, Veer Surendra Sai Institute of Medical Sciences and Research, Sambalpur, IND

**Keywords:** covid-19, sars-cov-2, pandemic, realtime reverse transcriptase - pcr, rt-pcr

## Abstract

Purpose

Right now, our world is in the grip of the COVID-19 pandemic. The global spread of COVID-19 (SARS-CoV-2) has dramatically increased the number of suspected cases with an expanding geographical area. The rapid identification of asymptomatic and mildly symptomatic contacts is the priority for clinical management and outbreak control. Suspected cases should be screened for the virus with a nucleic acid amplification test (NAAT) such as real-time reverse transcriptase-polymerase chain reaction (RT-PCR) under the guidance of laboratory experts.

Materials and methods

This manuscript details the process of the establishment of a COVID-19 lab, which is a medical college virology lab (Viral Research Diagnostic Lab), in less than a months' time. Detailed data of the tests were studied over the initial one month and reported.

Results

Within one and a half months of the start of the lab, 3196 tests were conducted, which caters to five adjoining districts in Western Odisha. These included both symptomatic and asymptomatic cases (contacts with a travel history from affected areas), and six COVID-19 positive cases were detected.

Conclusion

Though the establishment of a COVID-19 lab in a short time is a challenge, it can be achieved through determination, teamwork, and support from the authorities.

## Introduction

The COVID-19 pandemic has brought a halt to the world's progress. Scientists, doctors, and administrators are all perplexed and completely on the backfoot in their dealing with the situation. Countries have been locked down; an unforeseen situation.

Coronaviruses usually cause respiratory and gastrointestinal infections in humans and animals. However, three recently evolved coronaviruses, i.e., SARS-CoV, MERS-CoV, and now SARS-CoV-2 do cause severe respiratory illnesses in humans [1].

An agglomeration of pneumonia cases of unknown etiology was reported in Wuhan, China, in December 2019. Subsequent investigations identified a novel coronavirus as the etiologic agent [2]. It was initially named as the 2019 novel coronavirus (2019-nCoV) [2]. The virus was named SARS-CoV-2 by the International Committee of Taxonomy of Viruses (ICTV). The World Health Organization (WHO) refers to the virus as coronavirus disease 2019 (COVID-19) [3-4].

The coronavirus propagates through animals, human beings, and is also airborne, which spreads through infected respiratory droplets in cough and sneezes [3]. Clinical suspicion must be confirmed by laboratory diagnosis. Since the cases include both asymptomatic and symptomatic cases, finding them through contact tracing is important in the epidemiological point of view and to break the chain of spread.

WHO recommends the suspects to be screened using nucleic acid amplification tests (NAAT) such as reverse transcriptase-polymerase chain reaction (RT-PCR) [4]. Hence, setting up high-quality laboratories has been a priority for controlling the pandemicity of COVID-19.

## Materials and methods

Lab design

A lab area of approx. 227 sq.mt., was designed to have the requisite sections, namely:

Demarcated area for sample collection, sample storage, personal protective equipment (PPE) donning area, area for sample handling and ribonucleic acid (RNA) isolation, area for RT-PCR, demarcated area for doffing PPE, cold room, washroom, and all other electric backup systems [5].

Lab setup

Equipment in different sections, namely:

Biosafety level-2 plus laboratory (BSL-2), biosafety cabinet (BSC) class 2B, autoclave, automated RNA extractor, cold centrifuge up to 15,000 rpm for 1.5 ml tubes (for manual RNA extraction), vortex mixture, auto pipette set (1-5 µl, 2 µl, 10 µl, 200 µl, 1000 µl), water bath, incubator, -20°C deep freezer (for storage of extracted RNA, -80°C deep freezer (for storage of aliquoted samples), 4°-8°C refrigerator (to keep the viral transport medium until processing), calibrated real-time PCR machine with relevant detection channels, PCR work station, water purification system (MilliporeSigma, Burlington, Massachusetts), uninterrupted power supplies (UPS) 10 KVA - 2 hrs backup, and Spinwin centrifuge (Tarsons Product Pvt. Ltd., Kolkata, India) [5-6].

Manpower recruited at different sections per shift includes:

Medical microbiologists - two; Research scientist (non-medical) - one; Technicians - four for performing testing (including tutors and junior residents); data entry operator - two; Multipurpose worker (MPW) - two [6].

Each shift is for shift hours, and two shifts are in place.

Kits used

The RT-PCR kits used are MyLab Discovery Solutions (Pathodetect, Mylab Discovery Solutions Pvt Ltd., Maharashtra, India), SD Biosensor (nCoV Real-time detection kit, SD Biosensor, Republic of Korea), Seegene (Allplex 2019-nCoV Assay, South Korea) [5-7].

Manual RNA extraction was done using magnetic beads (BioFlux Extraction Kit, China), and automated RNA extraction is done using QIAcube (Qiagen, Hilden, Germany). Primers and probes were used as described [6,8].

**Figure 1:**
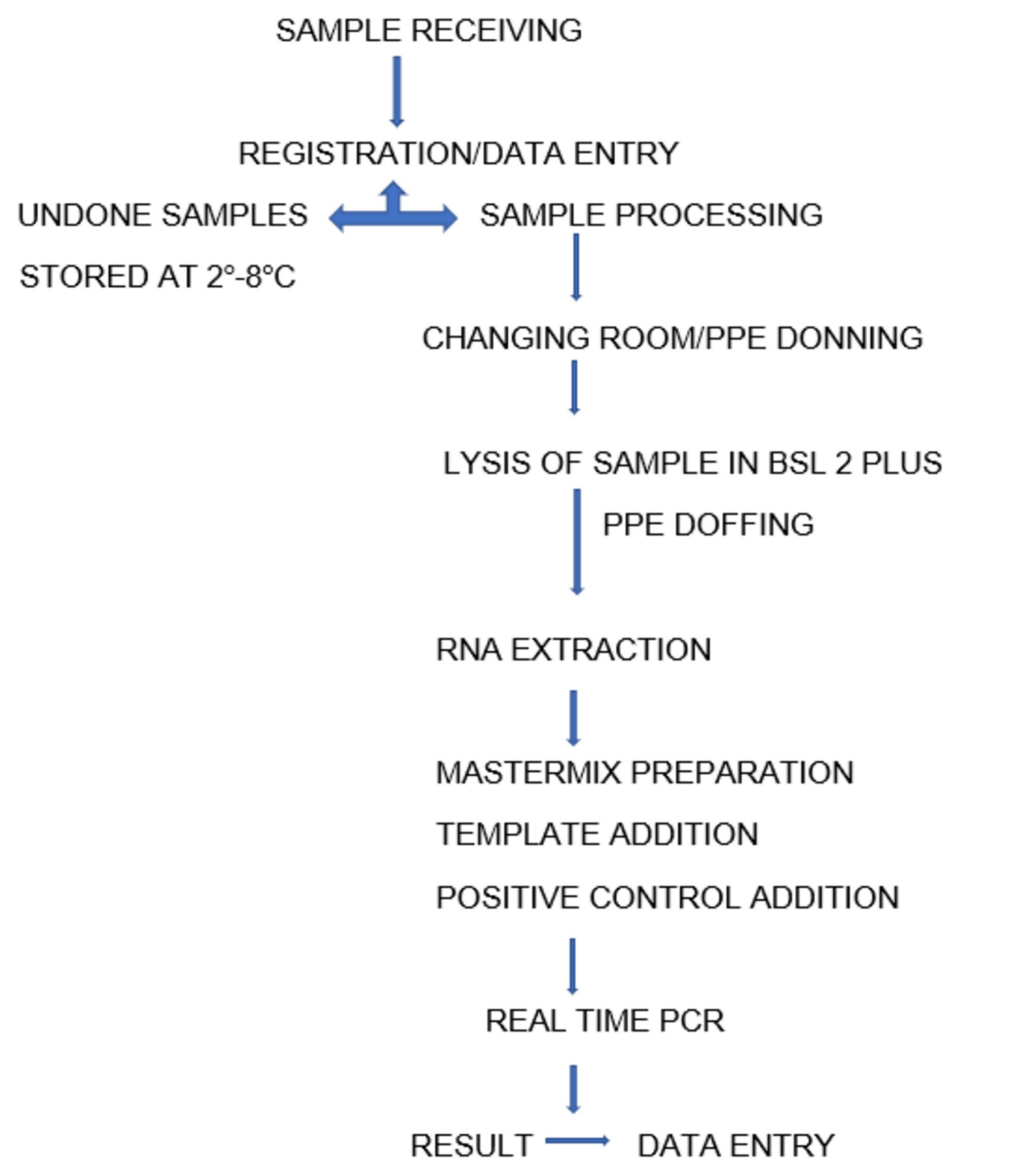
Flow chart of sample processing

The procedure from sample receipt to reporting is depicted in Figure 1 (Permission taken from the institute).

Stored samples were processed within 48 hours of sample collection.

## Results

**Figure 2:**
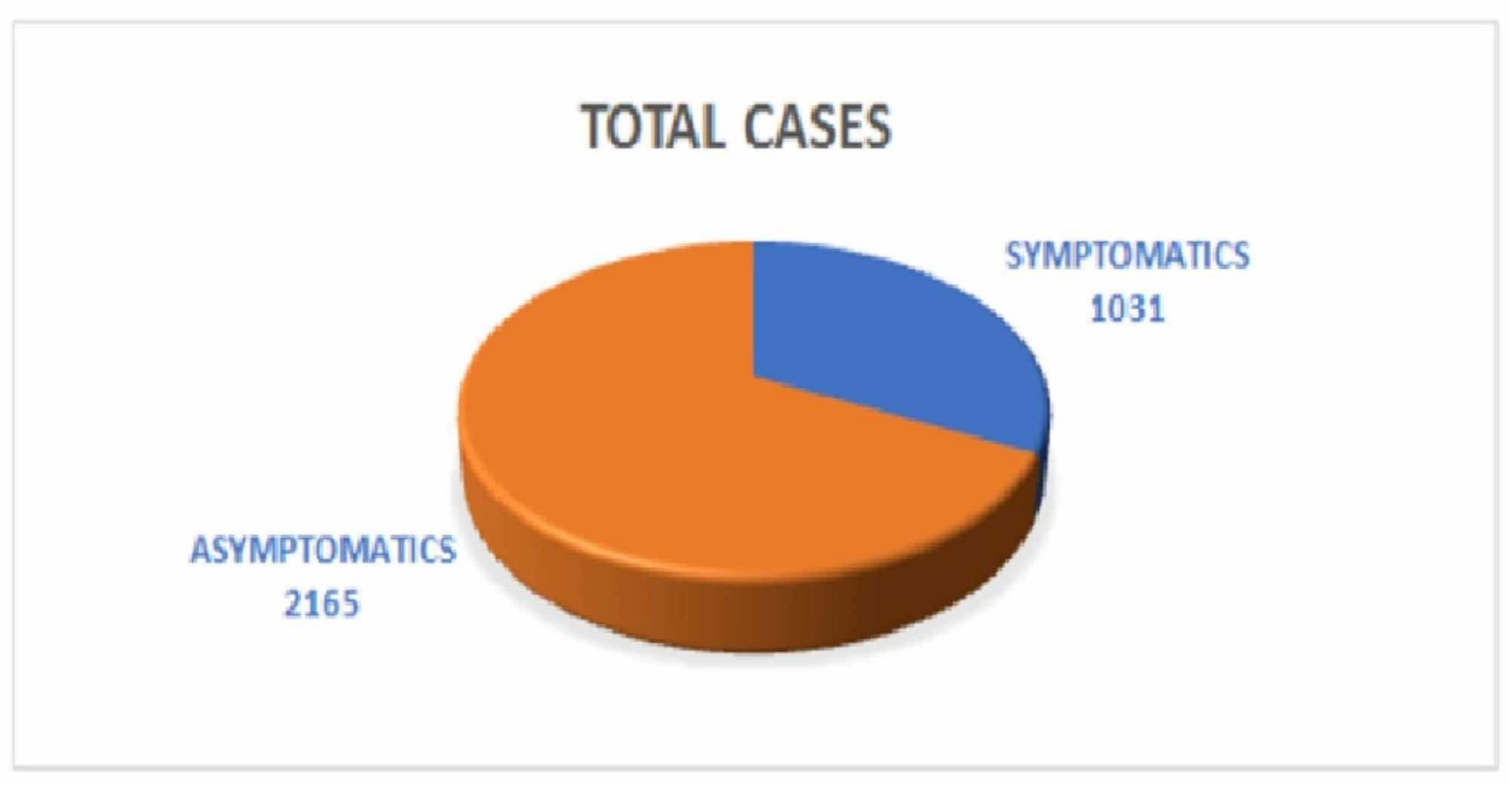
Total cases

Within one month of starting the lab, 3196 tests were conducted, catering to five adjoining districts in Western Odisha. This included symptomatic and asymptomatic cases (Figure 2). The age and sex distributions are shown in Tables 1-2.

**Table 1:** Age distribution of cases

AGE GROUP	NUMBER OF CASES
< 15 yr	83 (2.6%)
15-30 yr	682 (21.34%)
31-45 yr	435 (13.61%)
46-60 yr	824 (25.78%)
>60 yr	1172 (36.67%)

**Table 2:** Sex distribution of cases

SEX	NUMBER OF CASES
MALE	2002 (62.64%)
FEMALE	1194 (37.35%)

**Figure 3:**
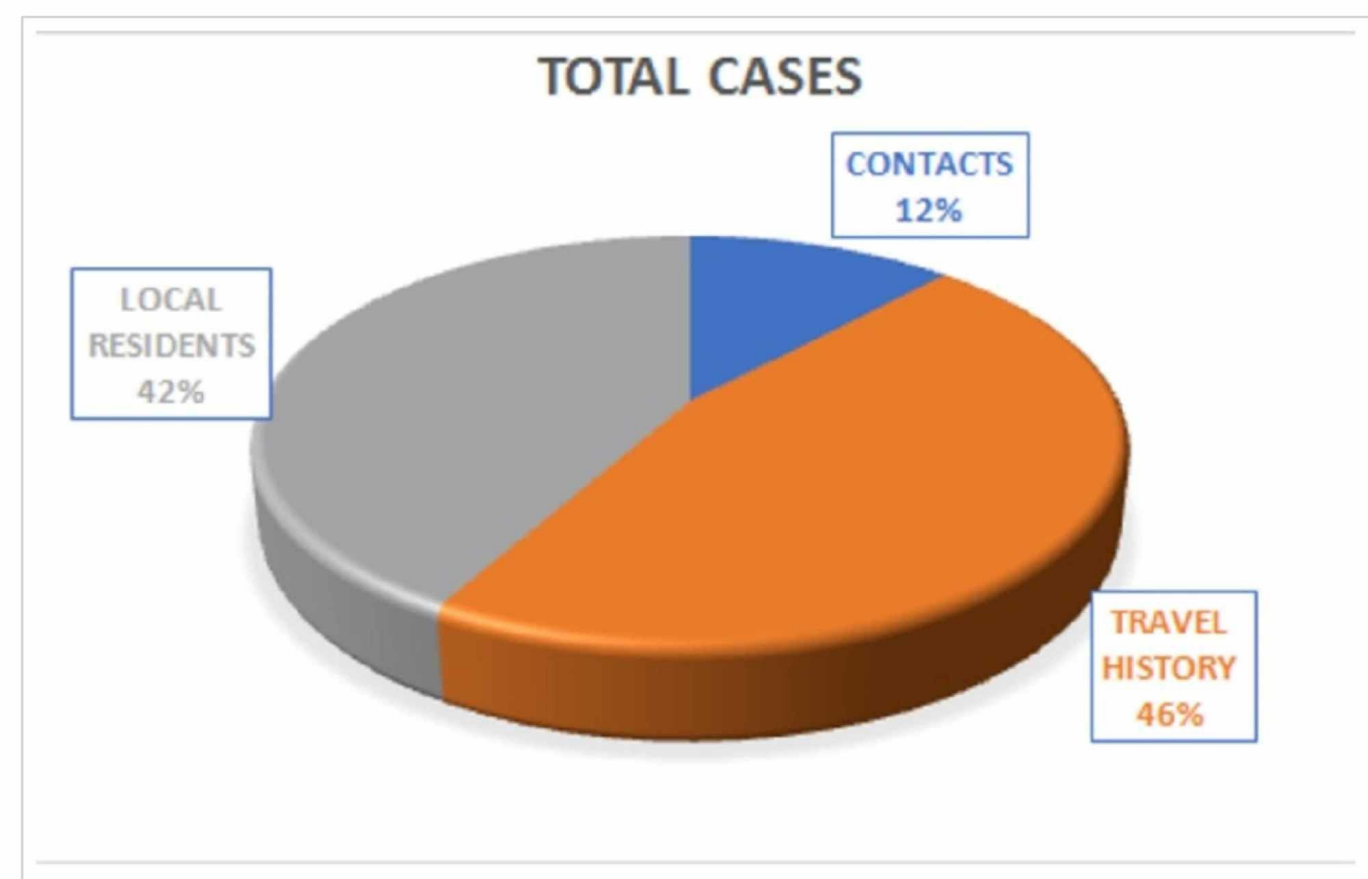
Types of cases

The total number of cases (3196) includes people with contact history, recent and past travel history (within the country and abroad) [9], and local residents. See Figure 3.

Six samples were rejected; one of them was had viral transport medium (VTM) tube leakage and five samples had a mismatch between ID number and the patient name labeled on the VTM tube [5].

**Figure 4:**
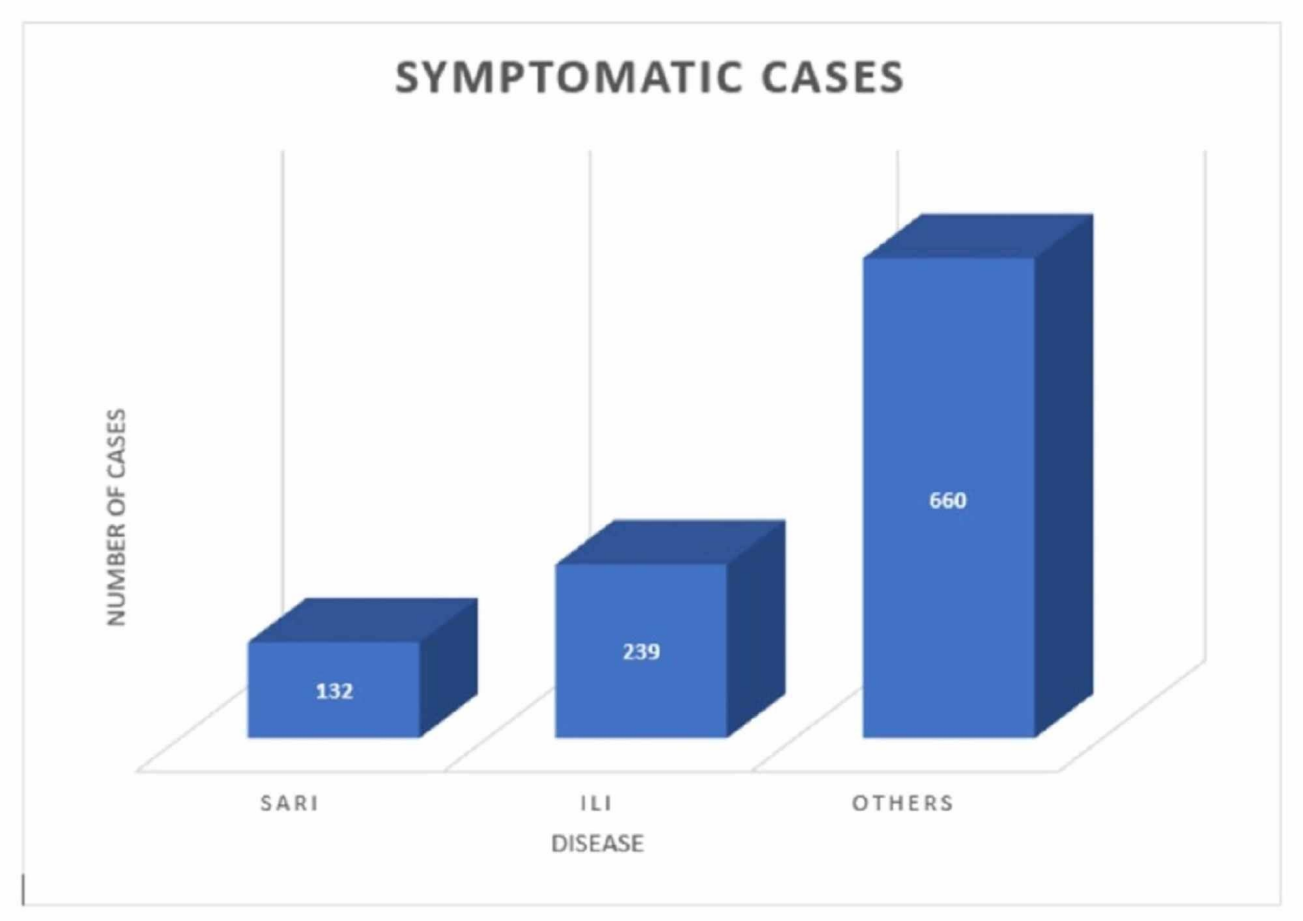
Symptomatic cases

The symptomatic cases (1031) are a combination of severe acute respiratory illness (SARI) [9], influenza-like illness (ILI) [10], and other symptoms like cough, sore throat, breathlessness, diarrhea, nausea, chest pain, nasal discharge, vomiting, fever, body ache, hemoptysis, sputum, and abdominal pain (Figure 4).

**Figure 5:**
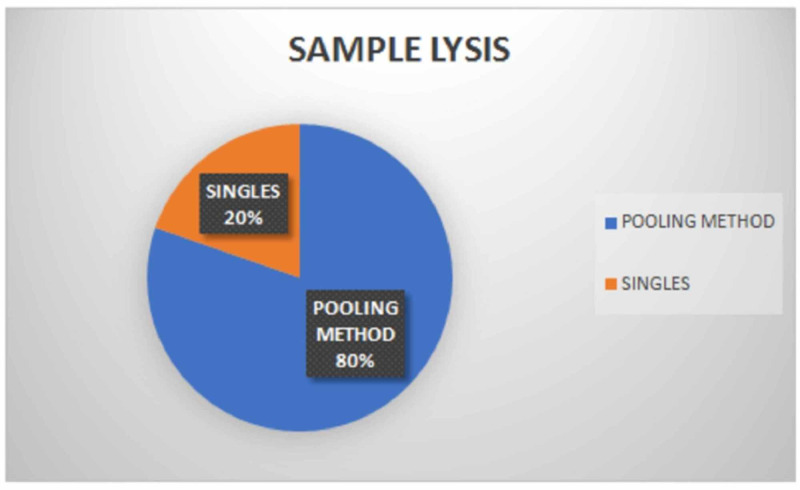
Sample lysis

All 3196 samples were processed (sample lysis) in two ways, i.e., 1348 samples were processed by pooling method [11] (pooling of 4 samples, total 337 pools) and 516 samples processed in singles (Figure 5).

**Figure 6:**
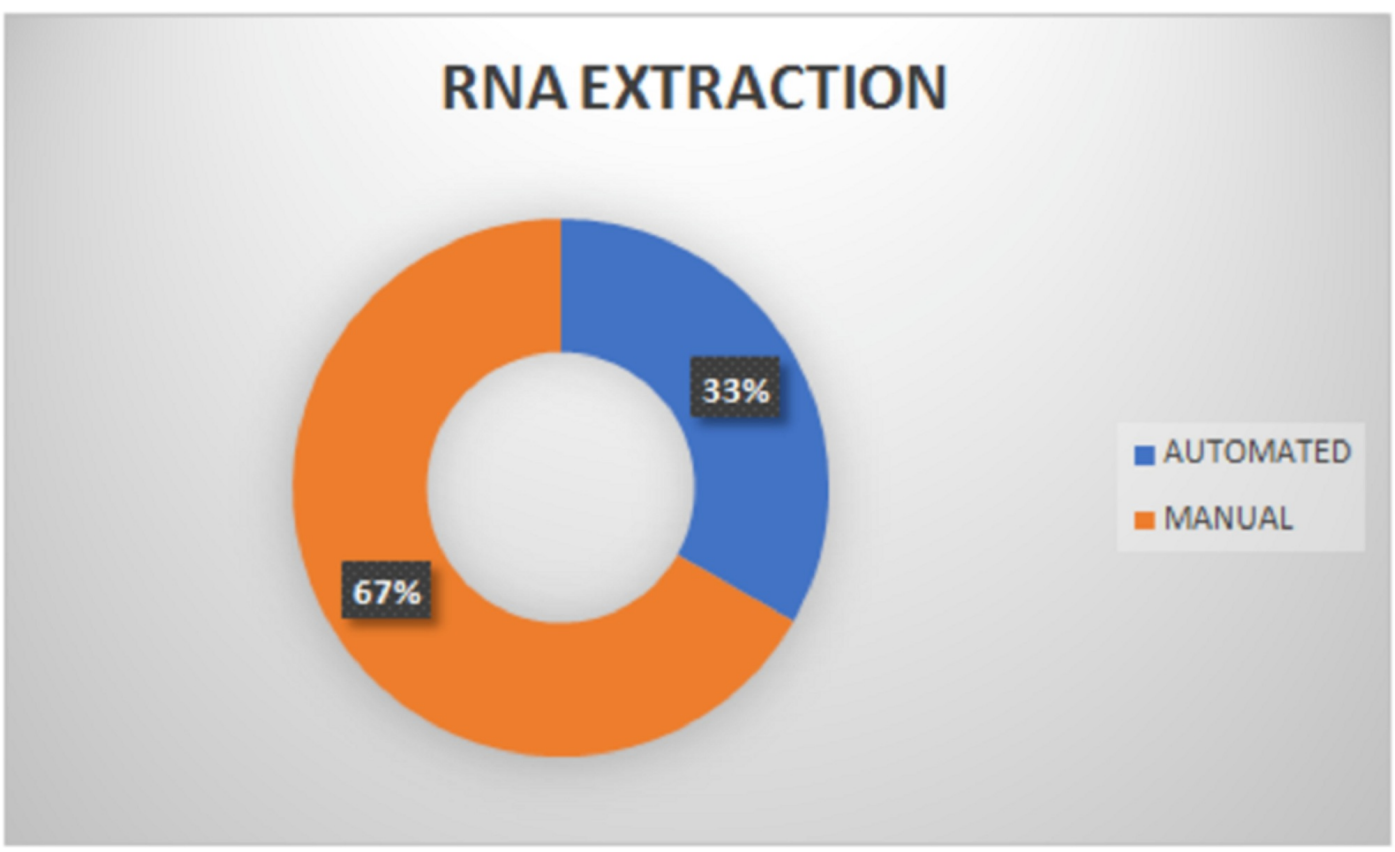
RNA extraction

Of then 853 lysed samples (337 pool of 4+516 single), RNA extraction was done by both automated and manual methods (Figure 6) [5,12].

Among the 3196 cases, six positive cases were detected.

Waste disposal

There were designated waste disposal areas in our lab [5,13-14], namely, sample collection room, PPE donning area, BSL 2 facility, RNA extraction/pre-PCR room, and PCR room. Double-layered bags are used for the collection of waste to ensure adequate strength and no leaks. The BSL 2 facility and PPE doffing area are labeled as“COVID-19 WASTE” to be identified as priority treatment and immediate disposal upon receipt. All the waste from suspected/confirmed patients are disposed of as medical waste into a double-layered medical waste bag, sealed with cable ties in a gooseneck fashion, and sprayed with 1% hypochlorite. All the waste from RNA isolation are treated with detergent and water and then with a 1% sodium hypochlorite solution. All dry wastes are discarded into a biohazard bag and autoclaved before disposal. The biomedical wastes are finally labeled as “COVID-19 waste” before handing over to the Common Biomedical Waste Treatment Facility (CBWTF).

## Discussion

In 1911, the Indian government set up the Indian Research Fund Association (IRFA) with the specific objective of sponsoring and coordinating medical research in the country [15]. After independence, several important changes were made in the organization and the activities of the IRFA. It was re-designated as the Indian Council of Medical Research (ICMR) in 1949, with a considerably expanded scope of functions [15]. At the end of 2016-17 (the 12th five-year plan), 82 Virus Research and Diagnostic Laboratory (VRDLs) had been approved and 29 VRDLs have become functional to carry out day-to-day diagnoses. During the 14th Finance Commission (2017 to 2019-20) the target was set to establish 60 more VRDLs [16].

The ICMR, the apex body of India for the formulation, coordination, and promotion of biomedical research throughout the country, in its guidelines dated April 8, 2020, laid down certain basic requirements pertaining to both infrastructure and expertise, as well as equipment and consumables, required to establish a real-time PCR testing facility to be fulfilled by the government and private medical colleges of India before applying to set up a COVID-19 testing facility in their facility [7]. In the first section describing the infrastructure and expertise, there are six points. A BSL-2 level laboratory facility should be available, which should include a molecular biology setup for virological diagnosis and functioning and calibrated Biosafety cabinet type 2A/2B. A cold centrifuge/microfuge to extract RNA should also be available. A functioning and calibrated real-time PCR machine should be at the facility's disposal. With respect to the manpower requirement, one or more medical microbiologists with experience in molecular virology, along with four to six technicians with experience in molecular virology who can work in two to three divided shifts and one or more multitasking staff to take care of washing/cleaning work. The medical microbiologist(s) and technicians should have a good understanding of laboratory biosafety and biosecurity. They must have been trained for handling respiratory samples for viral diagnosis, RNA extraction, and real-time PCR with an experience of work in virology and handling clinical specimens, especially respiratory samples. The institute must have its own time tested and a strong policy on biomedical waste management of human origin. At last, but not the least, there should be a well-defined arrangement for segregating and discarding biomedical waste. Private medical colleges should submit a National Accreditation Board for Testing and Calibration Laboratories (NABL) accreditation certificate and scope of accreditation for real-time PCR for RNA viruses.

The equipment required on-site for the laboratory includes a class 2A (calibrated) biosafety cabinet (BSC). A -20°C deep freezer for storing reagents (primers/probes/positive controls) and a -80°C deep freezer for the storage of aliquoted samples/viral RNA in cryovials both equipped with UPS.

 A refrigerator (for the storage of viral transport medium and for short-term storage of samples), two UPS each of 2 KVA capacity with two hours back-up, for real-time PCR instrument and nucleic acid extraction systems. The power backup for the two deep freezers should be confirmed. Finally, we require a real-time PCR machine, microcentrifuge/refrigerated centrifuge.

For sample collection, the requirements include PPE), VTM, and flocked dacron swabs (2 swabs/sample collection from one patient). The requirements during sample processing include a class IIA/IIB biosafety cabinet, PPE, a vortex mixer, a microcentrifuge (cold centrifuge), cryovials (2 ml), a cryobox, pipette aid, disposable plastic pipettes, forceps (if no spirit lamp, then disposable forceps for each sample), 70% ethanol (also required for extraction), 1% sodium hypochlorite, discarding jars, biomedical waste disposal (BMW) bags (with ties for sealing; preferably autoclavable) and bins, iceboxes with gel packs or regular ice supply in the laboratory, a tube rack (15 ml tubes) for tube/cryovial labeling - marker pens, cello tape, or label printouts.

For nucleic acid extraction, the laboratory should have manual extraction kits for viral RNA extraction, viral RNA mini kits (Qiagen) or other kits, 1.7 ml Eppendorf tubes (Hamburg, Germany), cryovial/Eppendorf tube rack, microcentrifuge, micropipettes - 100 to 1000 ul, 20 to 200 ul, filter barrier tips (1000 µl, 100 µl, 10 µl), tissue rolls, hand sanitizers, and biohazard labels.

The requirements for real-time PCR include a real-time PCR machine (open system) - calibrated for the fluorophore dyes that are present on the probes, reagents for setting up real-time PCR (primers and probes specific for SARS-CoV-2 targets and PCR master mix reagents with buffer and enzyme), PCR reagents (primers and probes for E gene screening and RNA-dependent RNA polymerase (RDRP)/ORF 1b targets, PCR buffer and enzyme mix, and positive control), one PCR workstation for master mix preparation and one for RNA addition, cryovial racks, PCR tubes, micropipettes - 0.5 µl to 10 µl (2 nos., 1 for PCR master mix and 1 for RNA addition), 2 to 20 µl, 20 to 200 µl, filter barrier tips - 10 µl, 100 µl, 1000 µl, a microspin (small equipment), plate centrifuge (small equipment), electronic micropipette (optional small equipment but convenient and reduces the time duration of testing), and nuclease-free water - for PCR and Ribonuclease P (RNase P). 

With the emergence of COVID-19 as a global pandemic, it became a necessity to establish more and more VRDLs in appropriate setups that match the above guidelines along with the existing laboratories for the correct, efficient, and timely diagnosis of the disease so that the necessary precautions can be taken to contain the further person-to-person spread of infection and treat the patient to save more human lives. Our institute, which is a tertiary care government medical college and hospital serving the people of Western Odisha, was selected as an appropriate center to establish a COVID-19 laboratory on March 28, 2020. Following the government’s proposal, a big hall in our department was selected exclusively as a COVID-19 lab. The necessary arrangements were done to procure the required equipment. The technical and non-technical staff, including the medical microbiologists working in the center (both faculties as well as postgraduate students ), underwent training for the use of PPE kits, sample collection, working in BSL-2, RNA extraction (both automated and manual), RT-PCR, biomedical waste management, and data entry. All training programs strictly followed the WHO and ICMR guidelines. The COVID-19 lab became functional on April 18, 2020.

According to the guideline, samples are collected from suspected patients [7], and tests were performed in appropriately equipped lab by staff trained in relevant technical and safety procedures. National guideline on lab biosafety was followed strictly [5,13-14]. Sample processing was carried out at BSL-2 wearing PPE following WHO recommendations [14]. Daily 100-130 samples were being tested. Samples received before 2 pm were analyzed on the same day and reported six and seven hours later to corresponding health authorities. Samples received after 2 pm were reported the next day. Rigorous preventive measures were taken to prevent contamination such as strict separation of working areas, dedicated PCR work station for PCR master mix preparation, and positive control addition in segregated rooms. All reagents were aliquoted, and aliquots were used only once.

Since its establishment, we have processed 3196 samples within one and a half months. We are trying to increase the number of samples tested per day, as the requirements are increasing daily as a greater number of suspected cases are being diagnosed daily. Any positive reports are sent to the All India Institute of Medical Sciences, Bhubaneswar, and the Regional Medical Research Centre, Bhubaneswar, for final confirmation before reporting to the State Health and Family Welfare Department. As of May 10, 2020, 340 government testing laboratories along with 131 private laboratories have become functional to speed up the testing capacity and early identification of suspected cases.

## Conclusions

The healthcare infrastructure before the emergence of the SARS-CoV-2 pandemic became overburdened after the unprecedented increase in the demand to test more and more samples rapidly and efficiently in a short span of time, as it was necessary to diagnose ad treat at the earliest. In the early half of March 2020, nationwide, there were only 52 laboratories authorized to carry out COVID-19 tests, now the number has grown up to over 25 times, with 1321 fully functioning laboratories out of which 907 are government-owned. Back in 2009, when the H1N1 swine flu pandemic spread across the world, India had only two viral research diagnostic laboratories capable of carrying out molecular virology tests. This gap in infrastructure prompted the Government of India in its collaborative efforts to set up newly designated laboratories. We conclude from the current scenario and our experience that we should be prepared beforehand to tackle another such unfortunate situation in the future by setting up new laboratories as per the population needs and to upgrade the present laboratories with state-of-the-art diagnostic modalities. The government should also plan to develop indigenous equipment to make India self-dependent and to decrease the economic burden on our healthcare system. Healthcare workers should be trained in the techniques by including it as a part of their curricula and conducting various workshops and continuing medical education (CME) programs at regular intervals to update their knowledge.
